# Copper and Nickel Induce Changes in the Lipid and Fatty Acid Composition of *Anodonta cygnea*

**DOI:** 10.3390/jox13010011

**Published:** 2023-03-01

**Authors:** Natalia Fokina

**Affiliations:** Institute of Biology, Karelian Research Centre of Russian Academy of Sciences, Pushkinskaya St., 11, 185910 Petrozavodsk, Russia; fokinann@gmail.com

**Keywords:** freshwater mussel, lipid response, unsaturated fatty acid, consequence, metal effect

## Abstract

The effect of copper and nickel ions on the lipid composition of freshwater mussels *Anodonta cygnea* was investigated using an aquarium experiment. The contents of the main lipid classes were determined using thin layer chromatography and spectrophotometry, and the fatty acid composition was analysed using gas–liquid chromatography. The results indicated that copper and nickel had different effects on the mussels’ lipid composition, with copper producing less effect on the composition of lipids and fatty acids than nickel. On the first experiment day, excessive copper content in the organism caused oxidative stress and modifications in membrane lipids, which returned to their initial level by the end of the experiment. Nickel accumulated predominantly in gills; however, significant modifications in lipids and fatty acids were seen also in the digestive gland from the first day of the experiment. This indicated the activation of nickel-induced lipid peroxidation processes. Moreover, this study revealed a dose-dependent effect of nickel on lipid composition, which was likely related to the development of compensatory biochemical mechanisms in response to nickel-induced oxidative stress. A comparative study of the lipid composition alteration in mussels in response to copper and nickel action revealed the consequences of the toxic impact of metal ions and the defensive mechanisms that organisms employ to detoxify and remove xenobiotics.

## 1. Introduction

Xenobiotics, including metal ions, that enter the body disrupt natural biological processes and trigger metabolic transformations in organisms [1]. Based on the type of their interactions with ligands (Lewis acidity), metals are classified into three classes: A, such as Na^+^, K^+^, Ca^2+^, and Mg^2+^, borderline, such as Mn^2+^, Zn^2+^, Ni^2+^, and Cu^2+^, and B, such as Cd^2+^ and Hg^2+^. Class A metals mostly form ionic interactions with ligands, whereas ions of the borderline and B classes form strong covalent bonds and heavily damage the structure of macromolecules and membranes [2]. However, some borderline class metals such as copper are vital for the organism. Copper is a cofactor for various enzymes and a component of hemocyanin [3,4,5]. Transition metals including copper can stimulate excessive formation of reactive oxygen species (ROS) and disrupt the balance of oxidation–reduction reactions, causing structural lesions in lipids, proteins, and DNA [3,6,7,8,9]. Unlike copper, nickel cannot generate ROS directly by participating in Fenton and Haber-Weiss redox reactions. However, nickel can induce ROS generation through a number of indirect mechanisms such as binding to macromolecules (proteins and nucleic acids) [10] and inactivating antioxidant enzymes [11]. ROS, whose main target is lipids, primarily polyunsaturated fatty acids (FAs) within membrane phospholipids, initiate lipid peroxidation (LPO) reactions [12,13]. Xenobiotics affect cells, causing damage to their structure and function, which may result in apoptosis and necrosis. Conversely, metal-induced LPO can lead to adaptive transformations in the antioxidant system [12,13] and the lipid profile of membranes, helping to stabilize their structure and permeability [14,15,16]. Owing to these defensive metabolic mechanisms, organisms can survive toxic xenobiotic impact.

Bivalves are used as bioindicators of pollution in monitoring studies, and for investigating the mechanisms of adaptation to environmental impacts at different levels of biological organization [3,8,12,15,17]. They accumulate organic and inorganic pollutants from water. The pollutants that accumulate in mussel tissues alter some of their biochemical parameters, reflecting the metabolic transformations designed to mitigate the xenobiotic impact. Existing literature describes multiple examples of the detrimental metabolic effects of metals in bivalves, specifically regarding the activity of antioxidant enzymes and LPO processes, such as changes in the concentration of conjugated dienes and malondialdehyde, activities in glutathione-S-transferase, catalase, etc. [3,10,12,13]. However, few studies deal with the metabolic implications of the toxic effect of metal ions on lipid composition. Moreover, lipids are actively involved in adaptation processes in aquatic organisms [18,19], and changes in the lipid profile shed light on the mechanisms for maintaining stability in organisms exposed to high anthropogenic pressure. The digestive gland is a reliable target-tissue for investigating the effects of xenobiotics in mussels at cellular, biochemical, and molecular levels [20]. Thus, examining the effect of the ion concentrations of different metals, such as copper and nickel, on the lipid composition of mussel digestive glands can reveal not only typical modifications indicating the metabolic disruptions caused by xenobiotic impact, including LPO activation consequences, but also specific modifications in the composition of lipids and FAs which support the adaptation process in bivalves. These results could help identify the potential biomarkers of metal ion stress in lipid composition.

## 2. Materials and Methods

### 2.1. Experiment Design

*Anodonta cygnea* (Linnaeus, 1758) mussels for the experiments were collected from the Suna river channel (Kondopozhsky District, Republic of Karelia, Russia) away from industrial pollution sources [19]. The metal content in the gills and digestive glands of *A. cygnea* mussels from the study area was trace and did not correspond to the values observed in contaminated areas [21,22,23].

Aquarium experiments for studying the effect of copper and nickel involved the control and maintenance of constant levels of the majority of hydro-chemical parameters: temperature, pH, dissolved oxygen concentration, total dissolved solids, total water hardness, anion composition (NH_4_^+^, NO_2_^–^, NO_3_^−^, SO_4_^2−^, and Cl^−^), and elemental composition.

A total of 160 mussel specimens, with shell lengths of 72.1 ± 6.4 mm, were used for each experiment. The mussels were acclimatized to laboratory conditions for seven days prior to the experiments. They were kept in aquariums with constant water temperature (22 °C) and pH (7.3), 12/12 photoperiod, and continuous water aeration. Water in the aquariums was partially replaced daily, and the water’s hydro-chemical parameters were controlled. These parameters remained relatively stable throughout the experiment. The animals were not fed while they were kept, in order to avoid changes in FA composition caused by an artificial commercial diet. After the acclimatization period, experiments were conducted in two replications for copper and nickel. The mussels were split into five groups: a control group kept in water without metal, and four experimental groups in water with the addition of 5, 50, 100, and 250 µg/L copper or 10, 50, 100, and 500 µg/L nickel. Stock solutions of metal salts were prepared by dissolving chlorides (Cu [II] chloride and Ni [II] chloride [hexahydrate]) in distilled water with a metal ion concentration of 20 mg/L. During the experiment, the water temperature, pH, DO, total mineralisation, total water hardness, and anion content were monitored daily and kept stable (mean ± SD): 21 ± 1.0 °C, pH = 7.0 ± 0.1, 7.8 ± 0.5 mgO_2_/L, TM: 11.8 ± 1.4 mg/L, TWH: 0.5–0.85 mmol/L, NH_4_^+^ 1.7 ± 0.4 mg/L, NO_2_^−^ 0.09 ± 0.03 mg/L, NO_3_^−^ 2.5 ± 0.0 mg/L, SO_4_^2−^ 14.2 ± 0.5 mg/L, and Cl^−^ 4.2 ± 0.4 mg/L. Analysis of the metal ion concentrations in the experimental aquarium water was conducted using the mass spectrometer XSeries-2 ICP-MS (Thermo Fisher Scientific, Cleveland, OH, USA).

After 1, 3, and 7 days of the experiment, the digestive glands of *A. cygnea* (n = 7 from each experimental group) were fixed in 97% ethyl alcohol and stored for no more than two weeks at 4 °C until biochemical analysis.

The soft tissues to be assayed for their cation content were frozen at −80 °C. After freeze-drying (Labconco FreeZone, Kansas City, MO, USA), they were analysed with an XSeries-2 ICP-MS mass spectrometer (Thermo Fisher Scientific, Cleveland, OH, USA). The powdered samples were digested in an acid mixture following the standard procedure [24].

### 2.2. Lipid Analysis

The lipid composition of the mussels’ digestive glands was examined using equipment at the Core Facility of the Karelian Research Centre RAS. Total lipids were extracted with chloroform/methanol (2:1 by volume) following the method developed by Folch et al. (1957) [25], using a Hei-VAP Advantage ML/G3 rotary evaporator (Heidolph Instruments, Schwabach, Germany). The contents of the main lipid classes were determined using thin layer chromatography and spectrophotometry. The extracted lipids were spotted onto silica gel thin-layer chromatography plates (TLC Silica gel 60 F254 plates, Merck, Darmstadt, Germany) and separated into different fractions of lipid classes using petroleum ether/diethyl ether/acetic acid (90:10:1, *v*/*v*) as the mobile phase. The identification of the fractions was performed using the following standards: phospholipid mixture (Sigma Aldrich, St Louis, MO, USA), cholesterol (Sigma Aldrich, St Louis, MO, USA), glyceryl trioleate (Sigma Aldrich, St Louis, MO, USA), and cholesteryl palmitate (Sigma Aldrich, St Louis, MO, USA). The quantitative composition of the fractions was measured at 540 nm wavelength for phospholipids, triacylglycerols, and sterol esters, and at 550 nm wavelength for the sterol fraction, using an SF-2000 UV/Vis spectrophotometer (Saint-Petersburg, Russia) [25,26,27]. The FA spectrum was analysed by gas–liquid chromatography using an Agilent 7890A system (Agilent Technologies, Palo Alto, CA, USA) with flame ionization detectors and DB23 capillary columns (Agilent Technologies, Palo Alto, CA, USA). The FAMEs were identified by comparing them with the standard mixes (Supelco, Bellefonte, PA, USA). The FA unsaturation index (FA USI) was estimated using the following formula: 

USI = (% sum of monoenoic FAs) + (2 × % sum of dienoic FAs) + (3 × % sum of trienoic FAs) + (4 × % sum of tetraenoic FAs) + (5 × % sum of pentaenoic FAs) + (6 × % sum of hexaenoic FAs)/% sum of saturated FAs.

### 2.3. Statistical Analyses

Statistical analyses were conducted using StatSoft Statistica v 7.0. As data distribution deviated from normality, as indicated by the Kolmogorov–Smirnov test, the significance of the differences was estimated using the non-parametric Kruskal–Wallis and Tukey’s post hoc tests. The reliability level was set at *p* ≤ 0.05.

## 3. Results and Discussion

**Figure 1:**
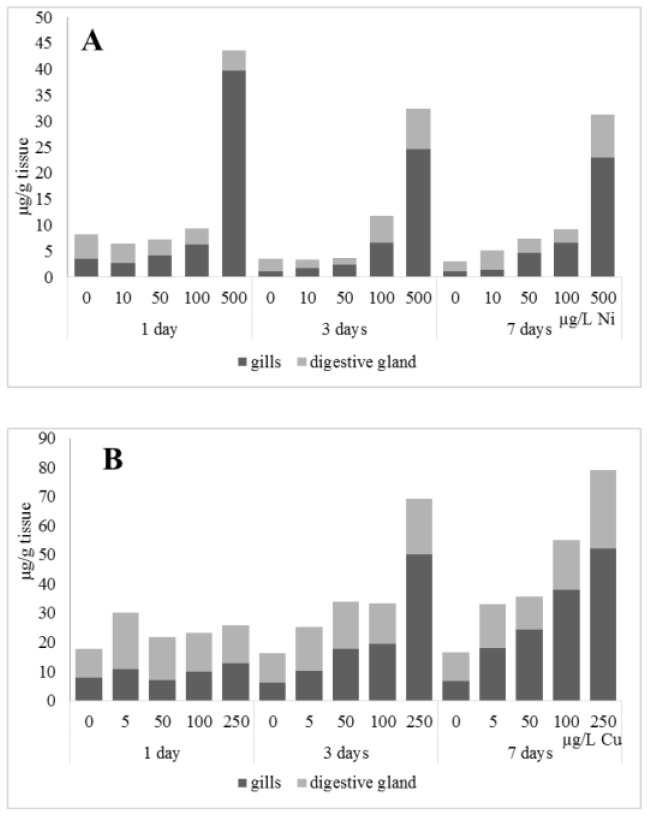
Content of Ni and Cu (µg/g tissue) in the gills and digestive gland of *A. cygnea* under nickel (**A**) and copper (**B**) experimental effects.

The accumulation of metals in mussel soft tissues, including copper and nickel intake efficiency, is influenced by the amount of dissolved metal and form it takes in the water (forming complexes with organic molecules), the animal species, and the filtering rate, which depends on the ambient temperature [28]. The locations for primary intake and accumulation of metals in freshwater mussels are the gills, mantle, and digestive glands, with the gills being the first to experience an increase in an increase in the content of the metals [10,29]. Experiments on the effects of nickel and copper on freshwater mussels demonstrated substantial accumulation of the metals in gills compared to digestive glands, as well as a deceleration of their accumulation in the soft tissues (Figure 1). This may suggest that *A. cygnea* have mechanisms to remove ions of these metals from the organism, with these mechanisms being disrupted due to high copper and nickel concentrations. The putative methods by which metals are excreted from mussels have been under-investigated. Similar dynamics of nickel and copper accumulation were demonstrated for the digestive glands of *Mytilus galloprovincialis* mussels; accumulation of the metals was relatively slow when concentrations were low (copper 0.2–20 mg/L, nickel below 100 µg/L), whereas exposure to 50 mg/L copper and nickel above 100 µg/L significantly promoted metal accumulation in mussel tissues [30,31].

### 3.1. Effects of Nickel

**Figure 2:**
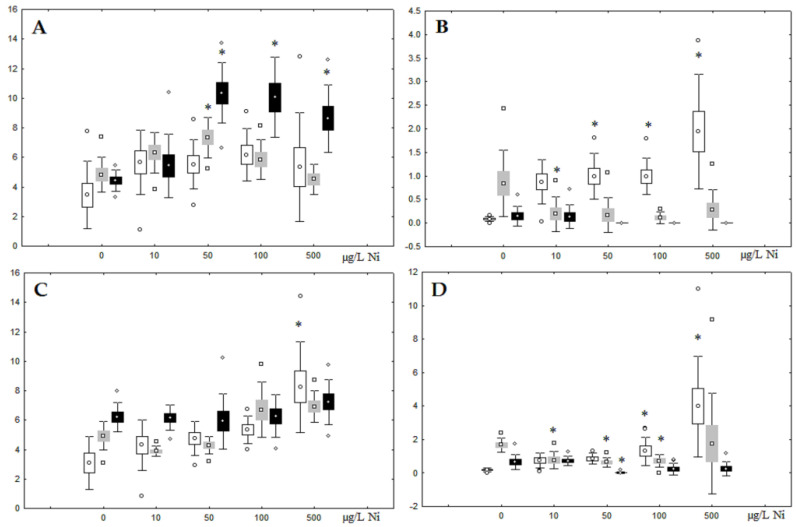
Content (% dry weight) of (**A**) phospholipids, (**B**) triacylglycerols, (**C**) cholesterol, and (**D**) cholesterol esters in the digestive glands of *A. cygnea* under experimental nickel effects: white box—day 1 of the experiment; grey box—day 3 of the experiment; black box—day 7 of the experiment. *—significant results in comparison with control (0 µg/L Ni). Differences were estimated using the nonparametric Kruskal–Wallis test; *p* < 0.05.

**Figure 3:**
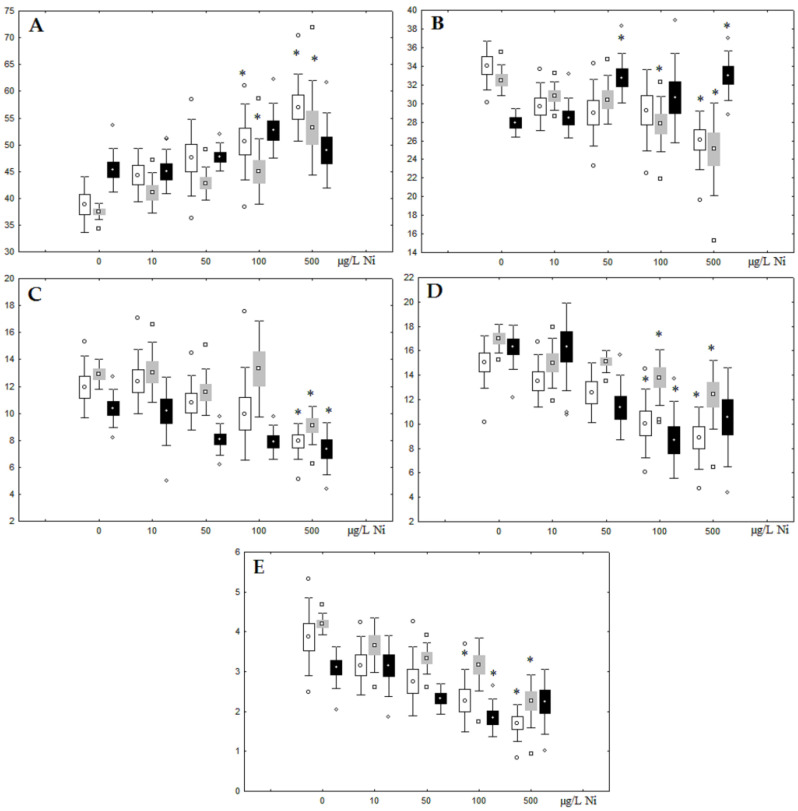
Changes in the (**A**) sum of saturated fatty acids, (**B**) monounsaturated fatty acids, (**C**) n-3 polyunsaturated fatty acids, (**D**) n-6 polyunsaturated fatty acids, and (**E**) unsaturation index in phospholipids of digestive glands of *A. cygnea* under experimental nickel effects: white box—day 1 of the experiment; grey box—day 3 of the experiment; black box—day 7 of the experiment. *—significant results in comparison with control (0 µg/L Ni). Differences were estimated using the nonparametric Kruskal–Wallis test; *p* < 0.05.

**Figure 4:**
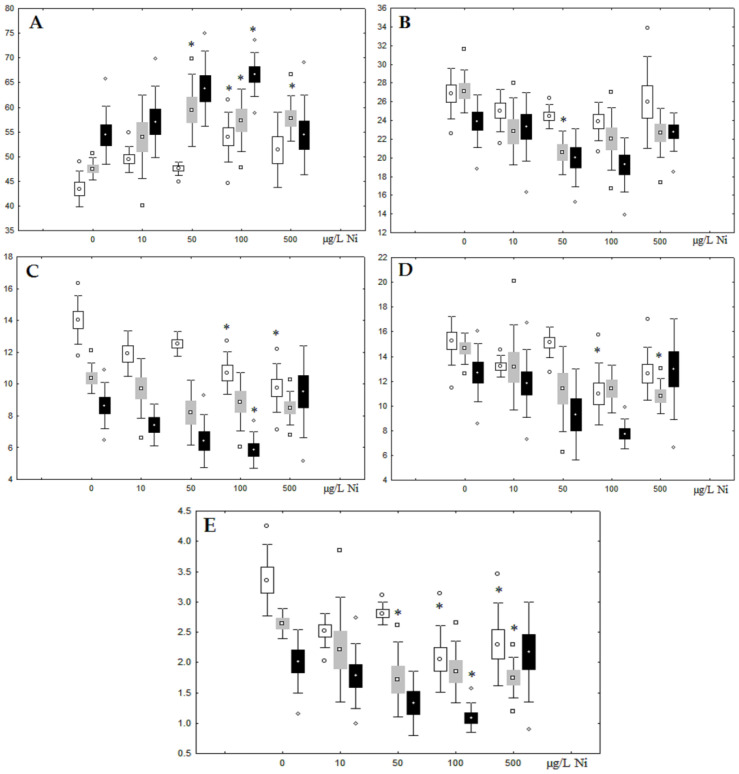
Changes in the (**A**) sum of saturated fatty acids, (**B**) monounsaturated fatty acids, (**C**) n-3 polyunsaturated fatty acids, (**D**) n-6 polyunsaturated fatty acids, and (**E**) unsaturation index in triacylglycerols of digestive glands of *A. cygnea* under experimental nickel effects: white box—day 1 of the experiment; grey box—day 3 of the experiment; black box—day 7 of the experiment. *—significant results in comparison with control (0 µg/L Ni). Differences were estimated using the nonparametric Kruskal–Wallis test; *p* < 0.05.

**Figure 5:**
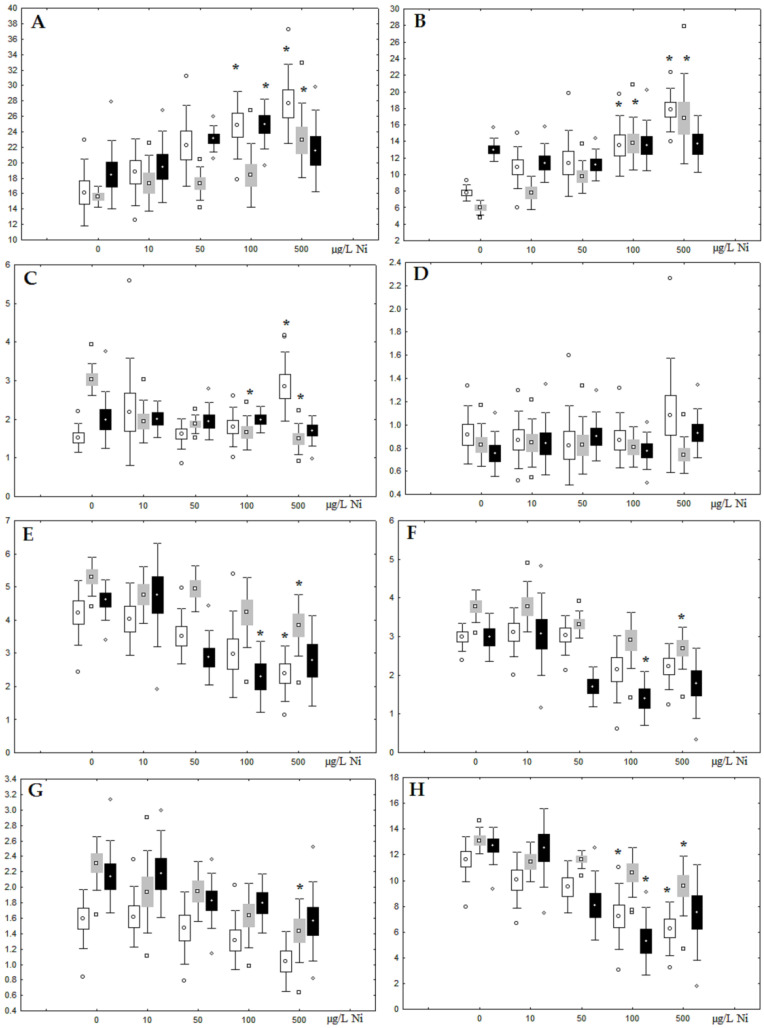
Changes in some dominated fatty acids in phospholipids: (**A**) 16:0, (**B**) 18:0, (**C**) 18:1n-9, (**D**) 20:1n-9, (**E**) 20:5n-3, (**F**) 22:6n-3, (**G**) 18:2n-6 and (**H**) 20:4n-6 in phospholipids of digestive glands of *A. cygnea* under experimental nickel effects: white box—day 1 of the experiment; grey box—day 3 of the experiment; black box—day 7 of the experiment. *—significant results in comparison with control (0 µg/L Ni). Differences were estimated using the nonparametric Kruskal–Wallis test, *p* < 0.05.

**Figure 6:**
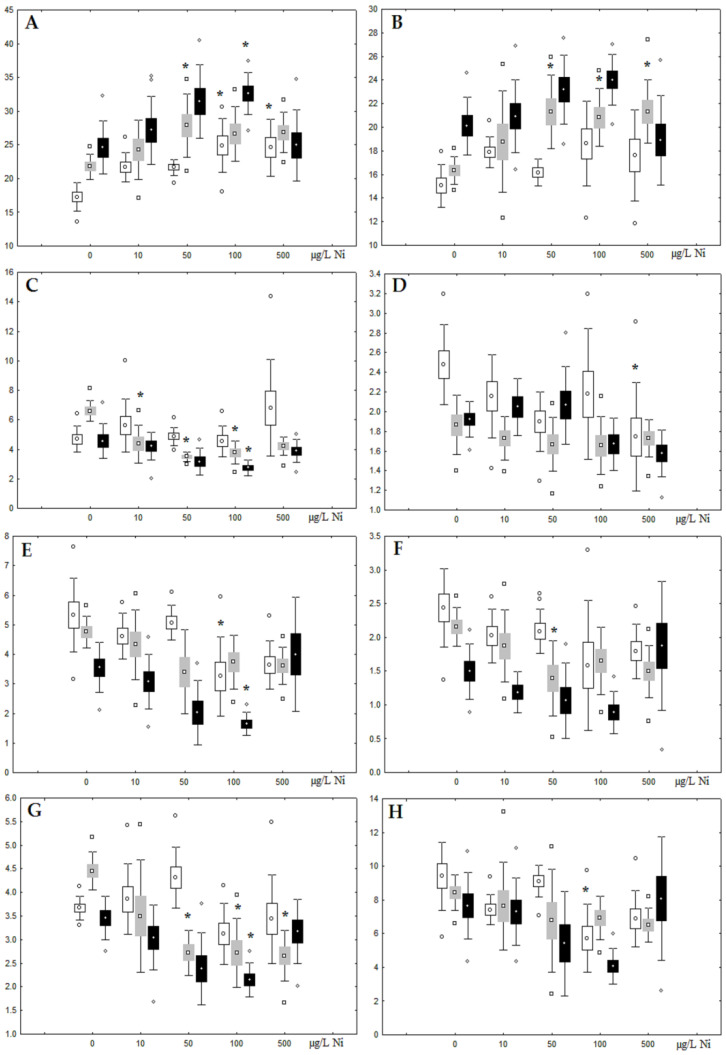
Changes in some dominated fatty acids in phospholipids: (**A**) 16:0, (**B**) 18:0, (**C**) 18:1n-9, (**D**) 20:1n-9, (**E**) 20:5n-3, (**F**) 22:6n-3, (**G**) 18:2n-6 and (**H**) 20:4n-6 in triacylglycerols of digestive glands of *A. cygnea* under experimental nickel effects: white box—day 1 of the experiment; grey box—day 3 of the experiment; black box—day 7 of the experiment. *—significant results in comparison with control (0 µg/L Ni). Differences were estimated using the nonparametric Kruskal–Wallis test, *p* < 0.05.

Although nickel accumulation in the digestive gland was minor (compared to the gills), we observed an increase in the contents of total lipids and triacylglycerols (TAGs) (Figure 2). Moreover, there was a reduction in the contents of monounsaturated FA (MUFA) and polyunsaturated FA (PUFA) in digestive gland TAGs (Figure 3, Figure 4, Figure 5 and Figure 6) on the first day of the experiment (mostly at 100 and 500 µg/L nickel). These findings indicate ongoing autophagy processes [32] and FA utilisation to meet the organism’s energy demands [18,33]. Autophagy activation is known to involve an increase in the concentration of neutral lipids, namely TAGs [32], whereas unsaturated FAs within TAGs are utilized to cover energy costs and are oxidized by ROS [18,33]. Further evidence of oxidative activity elevation, including LPO activation, in the experiments was the lowering of the FA USI and amount of n-3 and n-6 PUFAs in phospholipids (mostly at 100 and 500 µg/L) (Figure 3 and Figure 5). As the main target for oxidative processes within membrane phospholipids is PUFA [12,13,18,19], the lowering of the unsaturation level of FAs under xenobiotic impacts can be attributed mainly to the activation of lipid peroxidation [33]. Furthermore, we observed a significant increase in the content of cholesterol (Chol) and its esters, as well as of saturated FAs (SFA) within phospholipids (Figure 2, Figure 3, and Figure 5), indicating a compensatory stabilization of membrane structural organization [18,19] in response to the elevated activity of oxidative processes induced by nickel ion impact.

On the third day of exposure to the highest nickel concentration (500 µg/L), we observed changes in the composition of phospholipids (PL) and FAs (Figure 2, Figure 3, and Figure 5), similar to the observations on the first day of the experiment. The digestive glands exhibited a reduction in the contents of PL and USI (primarily due to low content of MUFAs, n-3, and n-6 PUFAs), as well as an elevated Chol content caused by nickel accumulation (Figure 2, Figure 3, and Figure 5). Oxidative stress under the impact of 50 µg/L nickel resulted in the lowering of MUFA contents and USI in TAGs (Figure 4 and Figure 6), whereas exposure to the lowest experimental concentration (10 µg/L) caused a compensatory reduction in TAG content and an increase in PL content (Figure 2). This appeared to be associated with detoxification processes, including compartmentalization, which is the formation of membrane vesicles such as metal-containing granules and autophagosomes [33,34]. 

On the first and third days of the nickel experiment, mussel gills [35] exhibited an activation of the FA metabolism, particularly unsaturated FA synthesis, which contributed to an increase in gill phospholipid unsaturation. Additional synthesis of unsaturated FAs (in particular, 20:1n-11 and 20:4n-6) in gill phospholipids was considered to regulate membrane viscosity and promote its permeability to ions. A high content of unsaturated FAs in mussel gills protects the membranes from oxidative destruction caused by these metal effects [35].

On the seventh day of the experiment, the mussels exhibited an increase in PLs in the digestive gland (50, 100, and 500 µg/L treatments; Figure 2). Changes in FAs were observed only under 100 and 500 µg/L nickel concentrations, and changes in the FA profile of TAGs depended on the metal concentration (Figure 4 and Figure 6). The effects of treatments with 100 and 500 µg/L nickel on the PL composition were elevation of the SFA level and lowering of 20:5n-3, 22:6n-3, and 20:4n-6 levels and the USI (Figure 3 and Figure 5), indicating oxidative stress damage to membrane structure. Within TAGs, exposure to 100 µg/L nickel induced an increase in 16:0, a reduction in MUFAs (18:1n-9, 20:1), a reduction in n-3 PUFAs (18:3 and 20:5), 18:2n-6, and USI, whereas the impact of 500 µg/L nickel caused a reduction in 16:0 and 18:0 and an increase in n-3 PUFAs (18:3 and 20:5), 18:2n-6, and USI (Figure 4 and Figure 6). The differences in the response of TAG FAs were due to the different levels of nickel ion accumulation in the digestive glands and, likely to the detoxification of the xenobiotics. Elevated nickel concentrations (100 and 500 µg/L) appear to disrupt the mechanisms of xenobiotic excretion and detoxification in the mussels, with significant consequences for the composition of lipids and FAs.

### 3.2. Copper Effects

**Figure 7:**
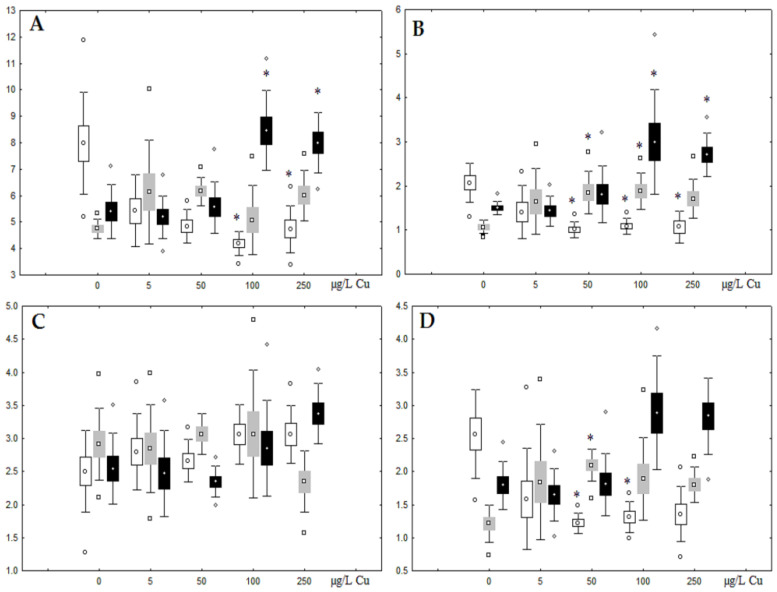
Content (% dry weight) of (**A**) phospholipids, (**B**) triacylglycerols, (**C**) cholesterol, and (**D**) cholesterol esters in the digestive glands of *A. cygnea* under experimental copper effects: white box—day 1 of the experiment; grey box—day 3 of the experiment; black box—day 7 of the experiment. *—significant results in comparison with control (0 µg/L Cu). Differences were estimated using the nonparametric Kruskal–Wallis test; *p* < 0.05.

**Figure 8:**
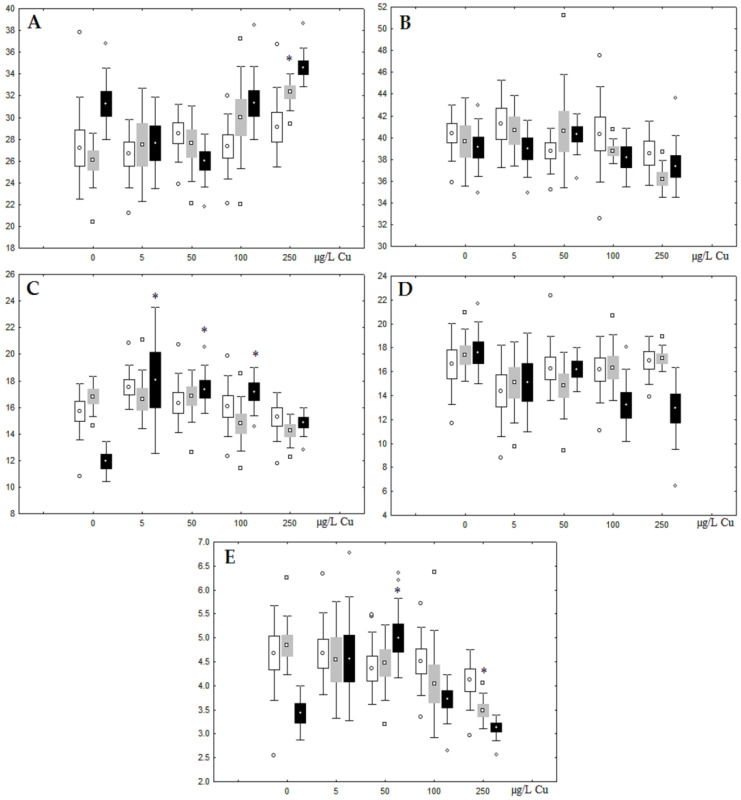
Changes in the (**A**) sum of saturated fatty acids, (**B**) monounsaturated fatty acids, (**C**) n-3 polyunsaturated fatty acids, (**D**) n-6 polyunsaturated fatty acids, and (**E**) unsaturation index in phospholipids of digestive glands of *A. cygnea* under experimental copper effects: white box—day 1 of the experiment; grey box—day 3 of the experiment; black box—day 7 of the experiment. *—significant results in comparison with control (0 µg/L Cu). Differences were estimated using the nonparametric Kruskal–Wallis test; *p* < 0.05.

**Figure 9:**
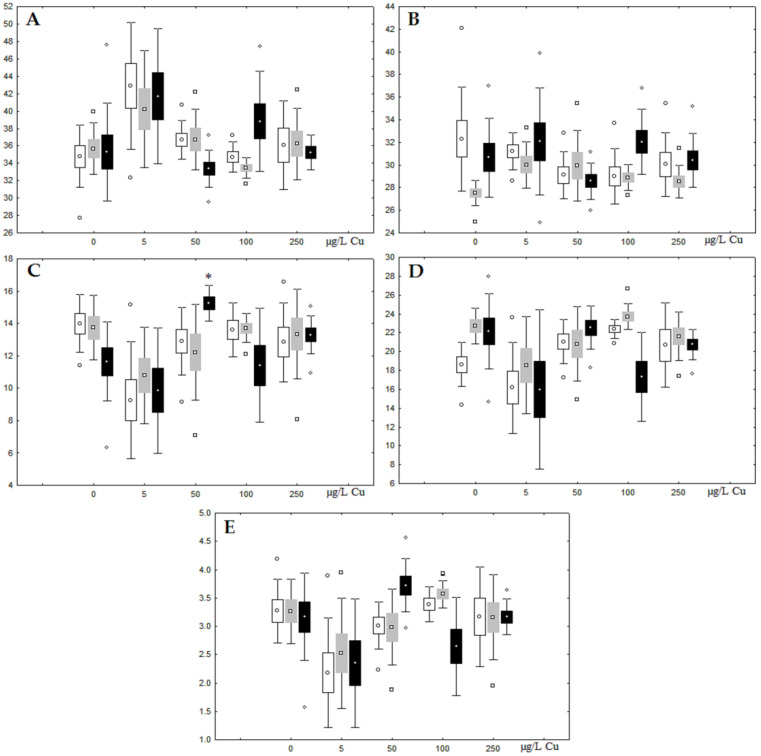
Changes in the (**A**) sum of saturated fatty acids, (**B**) monounsaturated fatty acids, (**C**) n-3 polyunsaturated fatty acids, (**D**) n-6 polyunsaturated fatty acids, and (**E**) unsaturation index in triacylglycerols of digestive glands of *A. cygnea* under experimental copper effects: white box—day 1 of the experiment; grey box—day 3 of the experiment; black box—day 7 of the experiment. *—significant results in comparison with control (0 µg/L Cu). Differences were estimated using the nonparametric Kruskal–Wallis test; *p* < 0.05.

**Figure 10:**
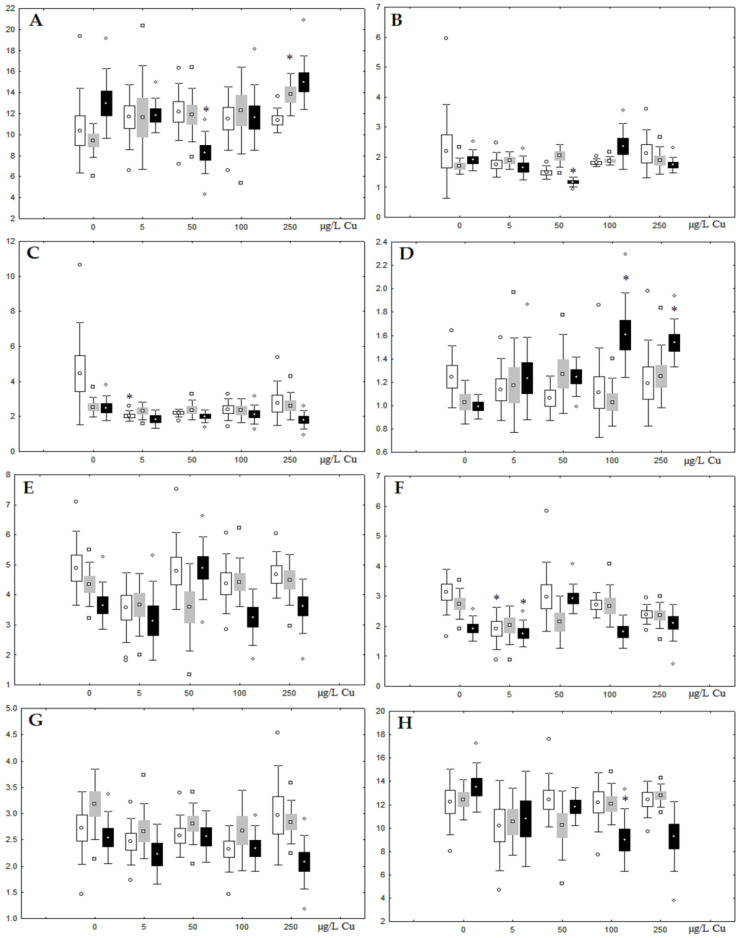
Changes in some dominated fatty acids in phospholipids: (**A**) 16:0, (**B**) 18:0, (**C**) 18:1n-9, (**D**) 20:1n-9, (**E**) 20:5n-3, (**F**) 22:6n-3, (**G**) 18:2n-6 and (**H**) 20:4n-6 in phospholipids of digestive glands of *A. cygnea* under experimental copper effects: white box—day 1 of the experiment; grey box—day 3 of the experiment; black box—day 7 of the experiment. *—significant results in comparison with control (0 µg/L Cu). Differences were estimated using the nonparametric Kruskal–Wallis test; *p* < 0.05.

**Figure 11:**
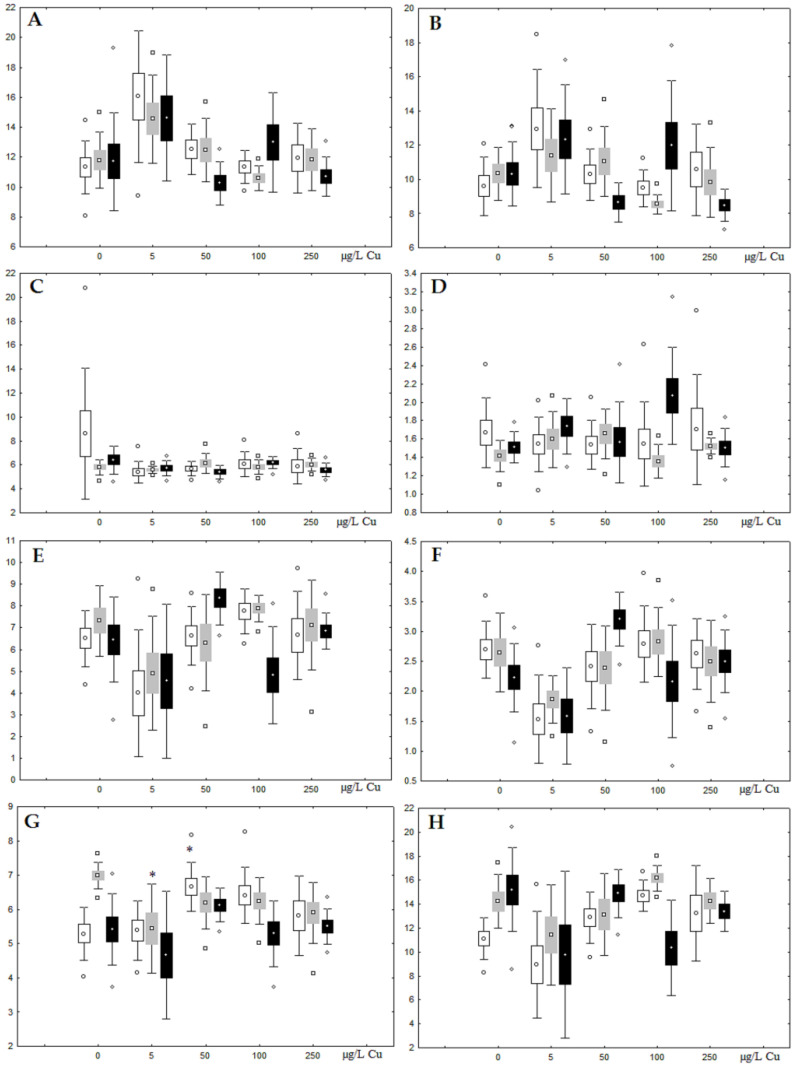
Changes in some dominated fatty acids in phospholipids: (**A**) 16:0, (**B**) 18:0, (**C**) 18:1n-9, (**D**) 20:1n-9, (**E**) 20:5n-3, (**F**) 22:6n-3, (**G**) 18:2n-6 and (**H**) 20:4n-6 in triacylglycerols of digestive glands of *A. cygnea* under experimental copper effects: white box—day 1 of the experiment; grey box—day 3 of the experiment; black box—day 7 of the experiment. *—significant results in comparison with control (0 µg/L Cu). Differences were estimated using the nonparametric Kruskal–Wallis test; *p* < 0.05.

Unlike nickel, we observed substantial copper accumulation in the tissues of the digestive gland on the first day of the experiment (Figure 1). Regarding lipid composition on the first day, all copper concentrations except for 5 µg/L, which was the lowest, induced a reduction in PL and TAG content and an increase in the content of Chol esters and the Chol/PL ratio (Figure 7). Furthermore, changes in PL and TAG FAs occurred only under the impact of the lowest concentration (5 µg/L), with reduced 18:1n-9 and 22:6n-3 contents in PLs and the n-3/n-6 PUFA ratio within TAGs (Figure 8, Figure 9, Figure 10 and Figure 11). Such dose-dependent effects of copper causing different modifications in membrane lipids was primarily associated with the rate of copper accumulation in the digestive glands and copper-induced oxidative stress. Changes on the third day of the experiment included an increase in the contents of TAGs (50 and 100 µg/L treatment) and Chol esters (50 µg/L treatment). Moreover, exposure to the highest copper concentration caused a reduction in the Chol/PL ratio, an elevation of the SFA level, and a lowering of the USI of FAs within PLs (Figure 7, Figure 8, Figure 9, Figure 10 and Figure 11). On the seventh day of the experiment, we saw an increase in the contents of PLs, TAGs, and Chol esters (100 and 250 µg/L treatments), as well as an increase in the content of n-3 PUFAs, some MUFAs and USI within PLs, and elevation of the n-3/n-6 PUFA ratio within TAGs. Thus, after seven days of copper impact, the lipid composition and FA profile were recovered from the oxidative stress induced by copper on the first day of the experiment. Furthermore, the gills exhibited an elevation in their level of unsaturated FAs, mostly during the first day of copper impact [36]. As unsaturated FAs perform a defensive function within membrane phospholipids [12,33], the mission of their additional synthesis in mussels experiencing copper-induced oxidation processes is likely to secure membrane integrity and permeability. Similar changes in PUFA levels were revealed in a study on the impact of copper on amphipods (*Dikerogammarus villosus*) and gastropods (*Helix pomatia*) [37,38,39]. On the seventh day of the experiment, gills contained elevated amounts of total lipids, chiefly membrane fractions (sterols and PLs), which was likely due to intensive formation of vesicles (metal-containing granules and autophagosomes) participating in compartmentalisation of the metals and their detoxification in the mussels [33,34]. The incomplete excretion of copper from mussels reportedly takes 10 days [40]. Active synthesis of membrane lipids apparently facilitates copper detoxification in mussels. By the end of the experiment (on the seventh day), we observed compensatory changes in the lipid composition of both gills and digestive glands, mostly underpinned by the detoxification function, that is, compartmentalisation and excretion of xenobiotics from the organism.

## 4. Conclusions

This study revealed significant differences in the response of mussels *A. cygnea* to the impact of two metal ions, copper and nickel, which are similar in the type of their interactions with ligands (Lewis acidity) and belong to the borderline ion class. Copper is an essential metal that is vital for the organism, whereas the biochemical effects of nickel’s impact on aquatic organisms are poorly understood [10,11,41,42]. This study demonstrated that as *A. cygnea* have acquired a mechanism for detoxifying and removing copper from the organism, copper produces less effect on the composition of lipids and FAs than nickel does. Nonetheless, although copper is an essential metal, its excess in the organism causes oxidative stress and modifications in membrane lipids, which recover to the initial status over time. The mechanism behind the toxic effect of nickel has rarely been examined; however, it is known to inactivate antioxidant enzymes and produce cytotoxic and genotoxic effects in molluscs [10,11]. The present results demonstrated that primary accumulation of nickel occurs in gills; however, significant modifications in the lipid and FA profile were also observed in the digestive glands from the first day of exposure, indicating an activation of lipid peroxidation. The dose-dependent effect of nickel on the lipid composition appeared to be related to disruption of the biochemical mechanisms for mitigating the consequences of oxidative stress with high nickel concentrations. Thus, a comparative study of the mussels’ lipid composition response to the impacts of copper and nickel would shed light on the consequences of the toxic impact of the metal ions, and detect its biomarkers, such as the FA unsaturation index in the lipid composition. Furthermore, our results revealed the defence mechanisms for detoxification and removal of xenobiotics from the organism and for maintaining resistance to high anthropogenic pressure. The Cu and Ni-induced modifications in the composition of lipids and FAs were specific to *A. cygnea*. These findings contribute to our understanding of the molecular mechanism of toxicity and the cellular defence mechanisms against these metals, which are involved in bivalves’ metabolic responses.

## Data Availability

Not applicable.
